# Biochar and Rhizobacteria Amendments Improve Several Soil Properties and Bacterial Diversity

**DOI:** 10.3390/microorganisms8040502

**Published:** 2020-04-01

**Authors:** Han Ren, Baoling Huang, Víctor Fernández-García, Jessica Miesel, Li Yan, Chengqun Lv

**Affiliations:** 1 Forestry College, Guangxi University, Daxue E Rd., Xiangtang District, Nanning 530004, Guangxi, China; renhan@st.gxu.edu.cn (H.R.); hnboln@126.com (B.H.); 13647886805@163.com (L.Y.); 2 Area of Ecology, Department of Biodiversity and Environmental Management, Faculty of Biological and Environmental Sciences, University of León, 24071 Leon, Spain; vferg@unileon.es; 3 Department of Plant, Soil and Microbial Sciences, Michigan State University, 1066 Bogue Street, East Lansing, MI 48824, USA; mieselje@msu.edu

**Keywords:** PGPR, physicochemical property, microbial composition, 16S rNA, *Bacillus megaterium*

## Abstract

In the current context, there is a growing interest in reducing the use of chemical fertilizers and pesticides to promote ecological agriculture. The use of biochar and plant growth-promoting rhizobacteria (PGPR) is an environmentally friendly alternative that can improve soil conditions and increase ecosystem productivity. However, the effects of biochar and PGPR amendments on forest plantations are not well known. The aim of this study is to investigate the effects of biochar and PGPR applications on soil nutrients and bacterial community. To achieve this goal, we applied amendments of (i) biochar at 20 t hm^−2^, (ii) PGPR at 5 × 10^10^ CFU mL^−1^, and (iii) biochar at 20 t hm^−2^ + PGPR at 5 × 10^10^ CFU mL^−1^ in a eucalyptus seedling plantation in Guangxi, China. Three months after applying the amendments, we collected six soil samples from each treatment and from control plots. From each soil sample, we analyzed several physicochemical properties (pH, electrical conductivity, total N, inorganic N, NO_3_^−^-N, NH_4_^+^-N, total P, total K, and soil water content), and we determined the bacterial community composition by sequencing the ribosomal 16S rRNA. Results indicated that co-application of biochar and PGPR amendments significantly decreased concentrations of soil total P and NH_4_^+^-N, whereas they increased NO_3_-N, total K, and soil water content. Biochar and PGPR treatments increased the richness and diversity of soil bacteria and the relative abundance of specific bacterial taxa such as Actinobacteria, Gemmatimonadetes, and Cyanobacteria. In general, the microbial composition was similar in the two treatments with PGPR. We also found that soil physicochemical properties had no significant influence on the soil composition of bacterial phyla, but soil NH_4_^+^-N was significantly related to the soil community composition of dominant bacterial genus. Thus, our findings suggest that biochar and PGPR amendments could be useful to maintain soil sustainability in eucalyptus plantations.

## 1. Introduction

Nowadays, there is a global challenge to find alternatives to reduce the massive use of chemical fertilizers and agrochemical products. In this sense, biochar and plant growth-promoting rhizobacteria (PGPR) are two eco-friendly alternatives that may be used to replace or reduce the use of these chemical products. Biochar has been reported as the product of high-temperature pyrolysis of organic matter in the absence or limited presence of oxygen. As a soil amendment, biochar has been shown to enhance soil quality, the efficiency of nutrient uptake by plants, and crop yield [1]. Biochar application leads to higher nutrient retention and nutrient availability via increasing the soil’s cation exchange capacity, surface area, and nutrient supply [2,3]. Charcoal amendments have also shown to have distinct effects on plant growth, including a greater development of the root system and higher yield [4]. However, we have found contrasting reports on the effects of biochar in soil physicochemical properties [5], nutrient contents [6], and in crop yield [1], suggesting that responses should be determined by biochar characteristics and soil types.

PGPR are a specific category of bacteria that bring benefits to plant growth and plant-microbe interactions [7]. PGPR improve plant growth by accelerating plant nutrient uptake and suppressing the adverse accumulation of soil microbes [8]. Plant growth-promoting using PGPR is exerted directly by providing essential nutrients and growth factors (phytohormone, Indole-3-acetic acid, cytokinins, etc.) to plants through biological nitrogen fixation, phosphate solubilization, and phytohormone production, and by reducing the biotic (i.e., plant diseases, insect pests, fungal infections) and abiotic (i.e., hydric and thermal fluctuations) stress indirectly for plant growth [8]. However, to our knowledge, previous studies have not clarified how PGPR amendments modify the composition of soil microbial communities. To address this gap it is important to know the effects of PGPR on soil biodiversity and soil processes and to further understand the underlying mechanisms allowing PGPR to act as an effective amendment via pathogenic bacteria inhibition.

Soil microbial diversity includes species diversity, genetic diversity, ecological diversity, and functional diversity, which provide evaluation criterion of the stability of the soil microbial community, soil quality, and soil biotic or abiotic stress [9]. Soil microbial diversity researching tools, such as the agar plate dilution method [10], Biolog EcoPlate method [11], molecular biology [12], or the metagenome and sequencing method [13], allow deep insights to be provided into microbial diversity and ecological processes in different environments [14,15]. Metagenomic sequencing methods have the potential to detect genes of microbial groups and germplasm, including the genetic information of cultured and uncultured microorganisms [13]. High-throughput sequencing, especially next-generation sequencing (Roche/454 FLX, Illumina/Solexa Genome Analyzer, and Applied Biosystems SOLID system) has been widely applied in soil metagenomics research and provides technical support and theoretical basis for uncultured soil microbial research [13].

Eucalyptus has become one of the most prominent pulps and wood raw materials in China since the 1970s because of its rapid growth, species variety, and resistance to various stresses. However, as an economically important plantation species, eucalyptus has also been widely reported to suppress soil microbial diversity, soil fertility, and ecosystem stability via rapidly depleting soil nutrients (especially nitrogen (N)) and water [16]. One approach to solving these problems is using bacterial fertilizer consisting of beneficial microorganisms and biochar through sustained biological N-fixing and reduced nutrition leaching [9,17]. 

Most studies of biochar and PGPR amendments aim at detecting their effects on plant growth, soil fertility, soil organic matter, and soil nutrition [4,6,18], rather than the relationship between soil nutrient content and soil microbial diversity. Even fewer studies have used the high-throughput sequencing method to address this issue [19]. In addition, it has also been reported that individual application of biochar or PGPR affects soil quality [20], soil water holding capacity [21], soil pH [22], plant vigor [23], crop yield [24], and soil microbial diversity [25], but the co-application of a mixture with biochar and PGPR as biofertilizer to benefit soil nutrient content and soil microbial diversity has not been studied well. It has been recognized that biochar can affect the soil and plant ecosystem in the short term [6,20], whereas the effects of PGPR on soil fertility and plant growth are manifested gradually over time [26]. Therefore, the objectives of our research were two-fold: (1) to determine the effects of biochar and PGPR amendments on soil properties over the short term compared to previous long-term studies and (2) to investigate the suitability of using a mixture of biochar and PGPR as new bacterial manure to improve soil quality and soil microbial diversity. To address this gap in the scientific knowledge, we specifically aimed to investigate the effects of co-application and individual application of biochar and PGPR on (i) soil nutrient concentrations, (ii) soil bacterial community diversity and composition, and (iii) the relationships between soil physicochemical properties and soil bacterial community composition. We hypothesized that the co-application of biochar and PGPR would increase soil nutrient concentration more than that of individual application of biochar/PGPR, potentially increasing soil microbial diversity.

## 2. Materials and Methods

### 2.1. Experimental Site

The experimental field is located in Nanning, Guangxi, China (107° 45’ 108° 51’ E, 22° 13’ 23° 32’ N). The average annual temperature at the research site is 21.6 °C from the year 2005 to 2015. The average annual rainfall is approximately 1300 mm, with an average humidity of 79%. At this site, the soil is classified as acidic metabolic red soil, with a pH in the range of 4.5–5.5 and a soil organic matter content of 2%–3% [18]. We selected eucalyptus seedling plantations for this study as they are the main crops in our study site, Guangxi being the most important producer of eucalyptus for wood in south China since the 1970s [27].

### 2.2. Biochar and PGPR Characterization

We used biochar made from wheat (*Triticum* L.) straw, produced in a continuous carbonizer at 600 ℃ for 3 h. The properties of biochar applied are shown in Table 1.

We used *Bacillus megaterium* de Bary, which is N_2_-fixing bacilli and a plant-probiotic species. *B. megaterium* strain DU07 was isolated from the eucalyptus rhizosphere in solid lysogeny broth (LB) in Liangfengjiang National Forest Park, Guangxi, China on June 2011 and then stored at −80 ℃ in an ultra-low temperature freezer for use. The strain was genotyped by sequencing part of the ribosomal 16S rRNA gene with the universal primers Y1 (5’-TGG CTC AGA ACG AAC GCT GGC GGC-3’) and Y2 (5’-CCC ACT GCT GCC TCC CGT AGG AGT-3’) by Shanghai Majorbio Bio-pharm Technology (Shangai, China). The record number of DU07 in GenBank at the NCBI (National Center for Biotechnology Information) was MK391000 [18]. 

Stored DU07 cells were cultured in fluid in LB at 28 °C under shaking at 120 r min^−1^ for 6 days for activation and were diluted to 5 × 10^10^ CFU mL^−1^ with sterile water before application.

### 2.3. Experimental Design and Soil Sampling

On January 2018, we established 12 plots of 10 m × 10 m, systematically separated using 2 m buffer strips, and allocated 3 for each of the following treatments: (i) biochar, (ii) PGPR, (iii) biochar+PGPR, and (iv) control.

(i) The biochar treatment consisted of digging holes of 30 cm × 30 cm × 30 cm and planting *Eucalyptus* seedlings (36 plants per 10 m × 10 m plot), filling the hole with a mixture of the extracted soil plus 0.18 kg biochar (corresponding to 20 t hm^−2^). (ii) The PGPR treatment consisted of digging holes of 30 cm × 30 cm × 30 cm and planting *Eucalyptus* seedlings (36 plants per 10 m × 10 m plot), with the extracted soil inoculated with 2 mL of the logarithmic-phase liquid culture of *B. megaterium* strain DU07. (iii) The biochar+PGPR treatment consisted of digging holes of 30 cm × 30 cm × 30 cm and planting *Eucalyptus* seedlings (36 plants per 10 m × 10 m plot), filling the hole with a mixture of the extracted soil inoculated with 2 mL of the logarithmic-phase liquid culture of *B. megaterium* strain DU07 plus 0.18 kg biochar (corresponding to 20 t hm^−2^). (iv) In the controls, holes were refilled with soil.

The *Eucalyptus* seedlings used for plantation were *Eucalyptus* DH32-29, a clone of Eucalyptus urophylla S.T. Blake × E. grandis Hill ex Maiden. Seedlings were bare-root, with a mean height of 25 cm, obtained from the Dongmen tree farm in 2018 (Guangxi, China). Roots were trimmed before planting.

Three months after planting the seedlings, we collected a soil sample from each 10 m × 10 m plot (3 samples per treatment) from the top 0–30 cm of the soil. Fresh soil samples were used to determine bacterial community diversity, and air-dried soil samples were used to analyze soil nutrient contents.

### 2.4. Analysis of Soil Physicochemical Properties and Bacterial Community

From each soil sample, we determined gravimetric soil water content (SWC), soil pH (water: soil = 2.5:1) with a pH-4 (Yidian, PHSJ-3F, China), and soil electrical conductivity (EC) with an EC-3 meter (Leici, DDSJ-308F, China). Soil inorganic N (NH_4_^+^-N and NO_3_^−^-N) and total N (TN) were determined in a flow injection auto-analyzer (Technicon, AA3, Germany) following digestion with H_2_SO_4_/HClO_4_ and NaHCO_3_ extraction. Soil total P (TP) was determined by the microplate method, and soil total K was determined via combustion in a flame photometer (Shuangxu, FP6430, China).

From each soil sample, we extracted microbial DNA using a E.Z.N.A.^®^ soil DNA Kit (Omega Bio-Tek, Norcross, GA, USA), according to the manufacturer’s protocols. The final DNA concentration and purification were determined using a NanoDrop 2000 UV-vis spectrophotometer (Thermo Scientific, Wilmington, DE, USA), and DNA quality was checked using 1% agarose gel electrophoresis. The V3-V4 hypervariable regions of the bacterial 16S rRNA gene were amplified with primers, as shown in Table 2, using a thermocycler PCR system (GeneAmp 9700, Applied Biosystems, Foster City, CA, USA). The PCR reactions program is shown in Table 3. The resulting PCR products were extracted from a 2% agarose gel and further purified using an AxyPrep DNA Gel Extraction Kit (Axygen Biosciences, Union City, CA, USA) and quantified using QuantiFluor™-ST (Promega Madison, WI, USA), according to the manufacturer’s protocol.

Purified amplicons were pooled in equimolar and paired-end sequenced (2 × 300) on an Illumina MiSeq platform (Illumina, San Diego, CA, USA), according to the standard protocols by Majorbio Bio-Pharm Technology Co. Ltd. (Shanghai, China). The raw reads were deposited into the NCBI Sequence Read Archive (SRA) database (Accession Number: SRP021124).

Raw FASTQ files were demultiplexed, quality-filtered using Trimmomatic, and merged using FLASH, with the following criteria: (i) The reads were truncated at any site receiving an average quality score <20 over a 50 bp sliding window. (ii) Primers were exactly matched allowing 2 nucleotide mismatching, and reads containing ambiguous bases were removed. (iii) Sequences whose overlap was longer than 10 bp were merged according to their overlap sequence. Operational taxonomic units (OTUs) were clustered with 97% similarity cutoff using UPARSE (version 7.1 http://drive5.com/uparse/), and chimeric sequences were identified and removed using UCHIME. The taxonomy of each 16S rRNA gene sequence was analyzed using the RDP Classifier algorithm (http://rdp.cme.msu.edu/) against the Silva (SSU123) 16S rRNA database using a confidence threshold of 70%.

### 2.5. Data Analysis

We calculated the bacterial α-diversity based on OTUs (operational taxonomic units). We used Chao1 (Equation S1) and ACE (Equations S2–S5) to characterize the richness of the bacterial community and Simpson (Equation S6) index to characterize the diversity of the bacterial community [28,29,30].

The effects of PGPR and biochar amendments on soil nutrient contents and bacterial community diversity were evaluated using ANOVA in R (http://www.R-project.org/). Where principal effects were significant, we used pairwise Tukey’s tests to determine significant differences among treatments. We conducted a regression analysis using the package Logistic Regression in R with statistical significance determined at α = 0.05. Redundancy analysis (RDA) and Monte-Carlo permutation tests were conducted using Canoco 5.0.

## 3. Results

### 3.1. Effects of Biochar and PGPR on Soil Physicochemical Properties

Both biochar and biochar+PGPR treatments increased concentrations of soil NO_3_^-^-N, inorganic N, electrical conductivity (EC), and soil water content (SWC), compared with the control (Table 4). B and PGPR significantly increased total N (TN), and soil total K (TK) was increased using PGPR and biochar+PGPR (Table 4). In contrast, soil total P (TP) and NH_4_^+^-N significantly decreased after B and/or PGPR treatments (Table 4). Additionally, we found significant differences in NO_3_^-^-N, inorganic N, TN, TP, TK when comparing the co-application and separate application of biochar and PGPR (Table 4). Soil pH was not affected by biochar and PGPR treatments (Table 4).

### 3.2. Effects of Biochar and PGPR on Microbial Richness and Diversity Indices

A total of 138,676 optimized sequences were obtained from sequencing (Table 5). The coverage index of soils amended with biochar and PGPR was between 98% and 99%, indicating that the dataset included all sequences between V2 and V3 regions and that sequence data volumes were reasonable.

The effects of biochar and PGPR on α-richness and α-diversity of bacteria based on OTUs are shown in Table 5. On the one hand, bacterial richness was positively affected by the co-application or separate application of biochar and PGPR, since all biochar and PGPR treatments significantly increased the ACE and Chao1 indices. On the other hand, biochar and biochar+PGPR treatments significantly (*p* < 0.05) increased the Simpson index in relation to the control. We also observed significant differences in the Simpson index between co-application and separate application of biochar and PGPR. PGPR significantly increased the bacterial diversity index relative to the co-application of PGPR and biochar, whereas the separate application of biochar showed the contrary trend.

### 3.3. Effects of Biochar and PGPR on Soil Bacterial Community Composition (Phylum Level) 

**Figure 1:**
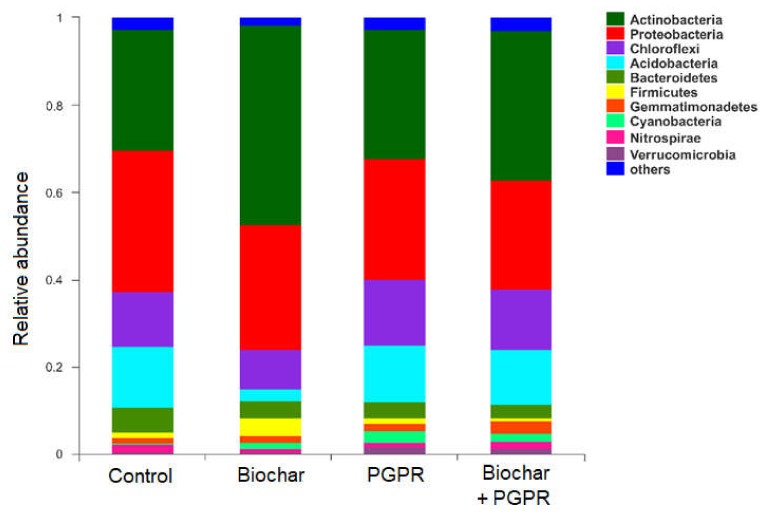
Relative abundances and community compositions of dominant bacterial phylum in soils for each treatment.

The analysis based on the 16S rRNA showed that the main bacterial phyla in soil samples were Proteobacteria (25.60%), Chloroflexi (19.10%), Actinobacteria (17.57%), Acidobacteria (9.65%), Bacteroidetes (6.89%), Planctomycetes (5.36%), Gemmatimonadetes (3.81%), Firmicutes (2.55%), Armatimonadetes (1.34%), and a relatively small amount (5.98%) of Verrucomicrobia and Spirochaetae and unclassified bacterial flora (2.15%) (Figure 1).

The bacterial composition of biochar and PGPR amended soils at the phylum level is shown in Figure 1. The relative abundance of Proteobacteria in biochar (0.29), PGPR (0.28), and biochar+PGPR (0.25) treatments was significantly (*p* < 0.01) lower than in the control (0.33). Significant differences between co-application and separate application of biochar and PGPR were also observed. A similar pattern occurred with Acidobacteria and Bacteroidetes, although we did not find significant differences between co-application and separate application of biochar and PGPR. On the contrary, the relative abundance of Actinobacteria was significantly (*p* < 0.001) higher in soils treated with biochar (0.46), PGPR (0.3), and biochar+PGPR (0.34) than in the control (0.28), and there were no significant differences between co-application and separate application of biochar and PGPR. A similar response was found for Gemmatimonadetes and Cyanobacteria. The abundance of Chloroflexi significantly (*p* < 0.001) increased in the PGPR treatment (0.15) compared to the control (0.12), but significantly decreased after biochar (0.09) and biochar+PGPR (0.01) treatments, and there were also significant differences between co-application and separate application of biochar and PGPR. The relative abundance of Firmicutes, Nitrospirae, and Verrucomicrobia after all or some of the biochar and PGPR was significantly higher than the control, and significant differences between co-application and separate application of biochar and PGPR were found.

### 3.4. Effects of Biochar and PGPR on Soil Bacterial Community Composition (Genus Level)

**Figure 2:**
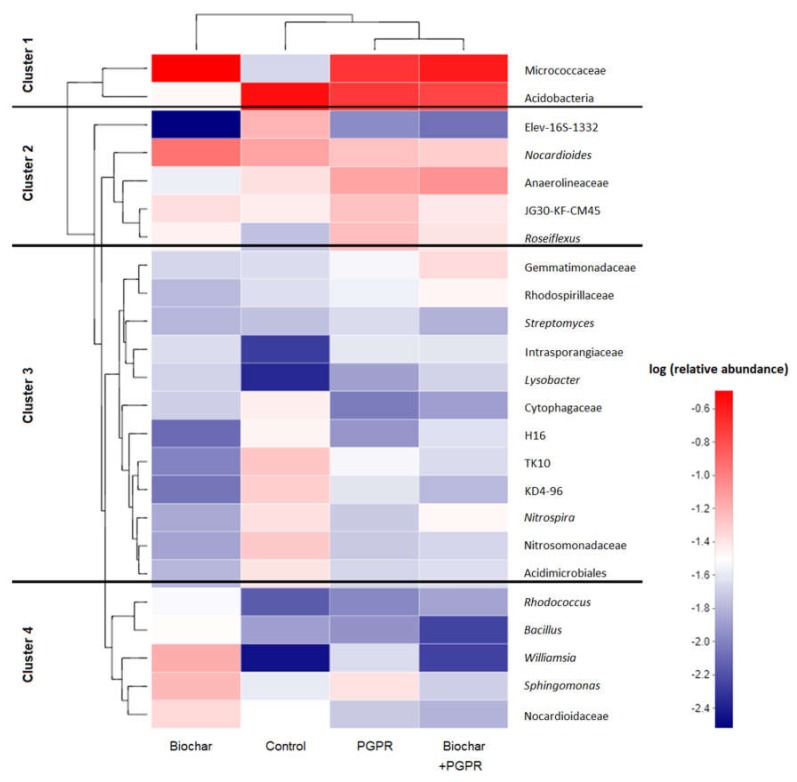
Relative abundances and community compositions of dominant bacterial genera in soils for each treatment. Their phylogenetic relationships are shown on the left tree. The top tree shows the cluster relationship among treatments.

We show the relative abundances and community composition of the dominant bacterial genera in soil via cluster analysis in a heatmap (Figure 2). The clustering result showed that biochar treatment was separately classified into a cluster (group 1), and control (group 2) was evidently separated from PGPR and biochar+PGPR treatments (group 3), indicating that the bacterial community of soils after PGPR amendments was significantly different than the bacterial community of soils after biochar treatment and the control. 

Soil bacteria genera were grouped into four clusters according to the abundance of each taxon (Figure 2). The most abundant genera were grouped in Cluster 1, composed of genera from Micrococcaceae and Acidobacteria. Genera with intermediate-high abundance were included in Cluster 2, in which the main genera of bacteria were from Nocardioides and Anaerolineaceae and the genus *Roseiflexus*. Intermediate-low abundant genera were included in Cluster 3, in which the main genera were from the families Gemmatimonadaceae, Rhodospirillaceae, and Intrasporangiaceae, and the genus *Streptomyces* and *Lysobacter*. Low abundant genera were included in Cluster 4, in which the main bacteria were the genus *Rhodococcus*, *Bacillus*, *Williamsia*, and *Sphingomonas*.

### 3.5. Correlations between Soil Physicochemical Properties and Soil Bacterial Community Composition

**Figure 3:**
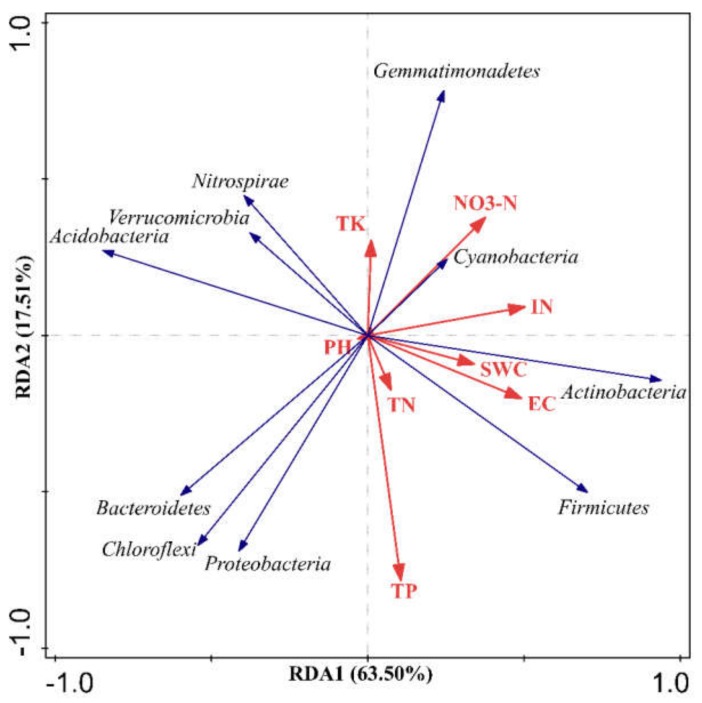
Redundancy analysis (RDA) of the composition of soil bacterial community at the phylum level and soil physiochemical properties. Bacterial phyla are represented by blue lines, and soil physiochemical properties (environmental factors) are represented by red lines. (TK: total potassium, NO_3_-N: nitrate nitrogen, IN: inorganic nitrogen, SWC: soil water content, EC: electrical conductivity, TN: total nitrogen, TP: total phosphorus).

**Figure 4:**
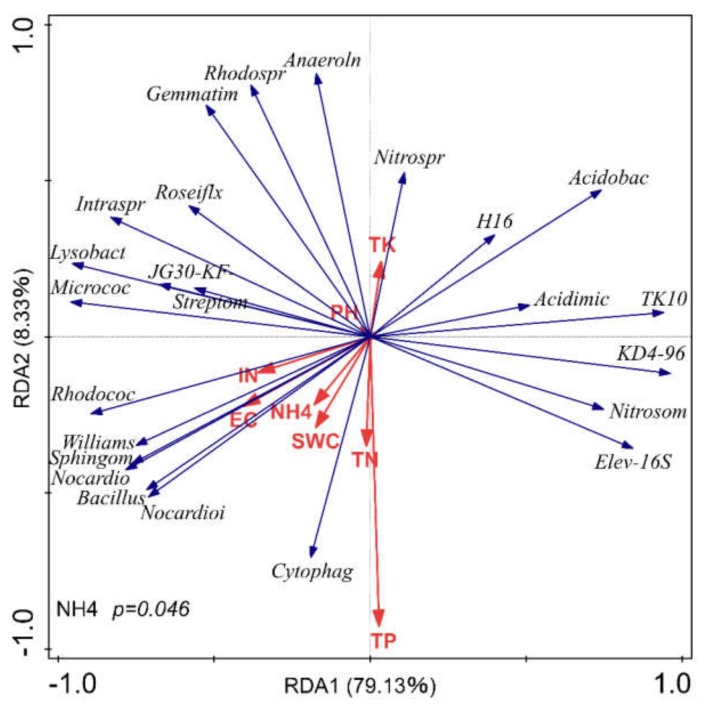
Redundancy analysis (RDA) of the composition of soil bacterial community at the genus level and soil physiochemical properties. Bacterial genera are represented by blue lines, and soil physiochemical properties (environmental factors) are represented by red lines. (TK: total potassium, IN: inorganic nitrogen, SWC: soil water content, EC: electrical conductivity, TN: total nitrogen, TP: total phosphorus; Anaeroln: Anaerolineaceae, Rhodosp: Rhodospirillaceae, Gemmatim: Gemmatimonadaceae, Roseiflex: *Roseiflexus,* Intraspr: Intrasporangiaceae, Lysobact: *Lysobacter,* Micrococ: Micrococcacea, Streptom: *Streptomyces*, Rhodococ: Rhodospirillaceae, Williams: *Williamsia,* Sphingom: Sphingomonas, Nocardioi: Nocardioidaceae, Cytophag: Cytophagaceae, Nitrosom: Nitrosomonadaceae, Acidimic: Acidimicrobiales, Acidobac: *Acidobacteria, Nitrospir: Nitrospira*).

The relationship between soil physicochemical properties and relative abundances of dominant bacterial was studied with redundancy analysis (RDA) at the phylum (Figure 3) and at the genus level (Figure 4). In general, the forward selection of RDA analyses showed that all physiochemical properties except NH_4_^+^-N affected soil bacterial community composition at the phylum level, whereas all soil properties expect NO_3_^−^-N affected soil bacterial community composition at the genus level, indicating differences in inorganic N preference among the different bacterial taxa.

The RDA of soil physicochemical properties and relative abundances of dominant bacterial phyla (Figure 3) show that the first ordination axis was correlated with Cyanobacteria, Actinobacteria and Firmicutes, and inversely related to Acidobacteria and Bacteroidetes, explaining 63.50% of the total variability. The second ordination axis was strongly related to Gemmatimonadetes and Proteobacteria, explaining 17.51% of the variability. The RDA revealed some trends; for instance, the relative abundance of soil Gemmatimonadetes was associated with TK and NO_3_^−^-N concentrations, Cyanobacteria with NO_3_^−^-N and TN concentrations, and Actinobacteria and Firmicutes with soil EC and SWC. However, the Monte-Carlo permutation test indicated that soil physicochemical properties were not significantly related to the bacterial community composition at the phylum level (inorganic N: *pseudo-F* = 2.00, *p* = 0.14; TP: *pseudo-F* = 1.50, *p* = 0.13; SWC: *pseudo-F* = 0.60, *p* = 0.52; TK: *pseudo-F* = 1.50, *p* = 0.27; NO_3_^—^N: *pseudo-F* = 2.70, *p* = 0.12; EC: *pseudo-F* = 1.90, *p* = 0.21; TN: *pseudo-F* = 2.50, *p* = 0.13; *pseudo-F* = 0.40, pH: *pseudo-F =* 0.31, *p* = 0.75).

The relationship between soil physicochemical properties and relative abundances of the dominant bacterial genera is shown in Figure 4. RDA revealed that the first ordination axis was strongly correlated with TK10, KD4-96, *Nitrosomonas*, and Elev-16S and inversely related to *Micrococcus*, *Lysobacter*, and *Rhodocuccus*, explaining 79.13% of the total variability. The second ordination axis was mainly associated with *Nitrospira,* Anaerolineaceae, and Rhodospirillaceae and inversely related to Cytophagaceae. RDA suggests that Nitrospira and Anaerolineaceae relative abundance is associated with TK content and inversely related to TP, TN, and SWC, whereas genera from the family Cytophagaceae showed the opposite pattern. Results of the Monte-Carlo permutation test indicated that soil NH_4_^+^-N was significantly related (*pseudo-F* = 6.50, *p* < 0.05) to the composition of the soil bacterial community at the genus level. The other soil properties were not significantly related to the community composition (inorganic N: *pseudo-F* = 3.20, *p* =0.10; TP: *pseudo-F* = 1.40, *p* = 0.3; SWC: *pseudo-F* = 0.60, *p* = 0.48; TK: *pseudo-F* = 0.70, *p* = 0.43; EC: *pseudo-F* = 1.50, *p* = 0.22; TN: *pseudo-F* = 0.20, *p* = 0.72; pH: *pseudo-F* = 0.70, *p* = 0.51).

## 4. Discussion

### 4.1. Effect of Biochar and PGPR on Soil Nutrient Content

This study determined the effect of PGPR and biochar on soil physicochemical properties. The increases in soil NO_3_^-^-N and inorganic N after biochar and biochar+PGPR treatments agree with other studies, as biochar could potentially absorb NO_3_^−^-N through the positive charge on biochar surfaces [31]. Biochar amendment could also alter soil water holding capacity and cation exchange capacity because of its large porosity and specific surface area [5], which is in line with the increased soil EC and SWC in biochar and biochar+PGPR treatments. PGPR application leads to the increased organic matter degradation rate, and thus increased soil soluble N compounds due to the high C/N [32], and then improved soil macro-nutrient concentration such as nitrogen [33], which is in line with the increased TN concentration in the PGPR treatment. The higher porosity, cation exchange capacity, and sorption capacity of charcoal may result in the accumulation of nutritive cations and anions [34], which is consistent with the increased TN in the biochar treatment. Our results also indicated that major increases in TK occurred in PGPR treatment, which can be attributed to the increased potassium solubilization capacity of the soil microbes [35].

In general, soil NH_4_^+^-N and TP concentrations significantly decreased in all biochar and PGPR treatments, suggesting that biochar application decreased the degradation of soil organic matter or biochar absorbed NH_4_^+^-N and soluble N and P compounds [32] in N-limited soils due to the high C/N. It is also possible that biochar amendments could interact with other soil environmental factors that influence NH_4_^+^-N and TP availability, such as the diversity of the soil microbial community, the rates of nutrient mineralization, or changes in soil texture that may influence nutrient retention. Furthermore, biological N-fixation and P-solubilization by PGPR is a long-term process, whereas soil nutrient uptake may also occur due to a large number of rhizobacteria being applied, which may explain why PGPR application decreased soil NH_4_^+^-N and TP in the short term in our study.

For soil physicochemical properties, co-application of biochar and PGPR significantly increased soil NO_3_^-^-N, inorganic N, and TK in relation to that of separate application of biochar and PGPR. When biochar and PGPR were co-applied as a soil amendment, the soil fertility increased to a relatively high level, potentially followed by biochar accelerating the conversion of soil NH_4_^+^-N to NO_3_^−^-N for soil N retention [20], and thus leading to the significant increase in co-application of biochar and PGPR. Soil inorganic N increased with co-application of biochar and PGPR relative to separate application. This effect agrees with the widespread assumption that PGPR increases nitrogen fixation [33] and that biochar leads to reduced nitrogen leaching [36]. Significant increase of soil TK in co-application of biochar and PGPR compared with separate application of biochar/PGPR has also occurred, the main reason likely being that biochar is difficult for mineralization [36], whereas it easily absorbs potassium (K^+^) [37] from K-solubilization using PGPR in the topsoil and may lead to decreased K loss.

### 4.2. Soil Microbial α-Diversity Indices

Our results showed that biochar and biochar+PGPR significantly increased soil bacterial diversity (Simpson index) and richness (ACE and Chao1 indices). One of the factors that may affect the diversity of the soil bacterial community is soil acidity [38], which is slightly increased by biochar in our study. The increased soil microbial richness may be a result of improvements in the soil environment from biochar and PGPR separately and co-applied, such as enhancement of soil structure, inorganic and organic nutrition input, or higher water holding capacity [8,39]. Changes in these environmental factors may accelerate the metabolism and reproduction of microbial communities, and thus elevate soil bacterial richness [40]. PGPR significantly increased the bacterial diversity index relative to co-application of PGPR and biochar, whereas separate application of biochar showed the contrary trend. Soil organic matter content and TN are indicators of potential soil nutrition status, as well as soil bacterial community diversity [19]. Hence, the relative higher diversity (Simpson) and richness (ACE and Chaol) in the PGPR separate application treatment may be a result of sufficient N supply from the biological N-fixing using the strain DU07 amendment. On the contrary, Rondon et al. [41] reported that biochar application may reduce the utilization capacity of phenolic acids carbon sources by bacteria, potentially decreasing diversity of the soil bacterial community.

### 4.3. Soil Bacterial Community Composition (Phylum Level)

The Proteobacteria phylum is one of the most diverse and fastest metabolics in bacteria, and it mainly plays a part in maintaining soil ecological stability via soil nitrogen supply [42]. Acidobacteria is mainly distributed in the terrestrial environment, ocean and activated sludge, demonstrating general adaptability and functional diversity [23]. In the short term of PGPR application, limited available nitrogen could be supplied to bacterial growth and reproduction as biological N-fixing by PGPR was in a time-release manner [18], which is consistent with the decreased relative abundance of Proteobacteria, Acidobacteria, and Bacteroidete in PGPR treatment. Rondon et al. [41] reported that P became the limiting factor in an N-sufficient soil for microbial growth after biochar was applied. In our study, 20 t hm^−2^ biochar significantly increased soil NO_3_^−^-N, inorganic nitrogen, TN, and TK concentrations; however, the decreased soil TP concentration in the biochar separate application treatment may become the limiting factor for soil Proteobacteria, Acidobacteria, and Bacteroidetes metabolism and reproduction [43].

Actinobacteria belongs to gram-positive bacteria, and it could degrade cellulose and chitin as the main resource for soil nutrient supply. Huang et al. [44] reported that soil Actinobacteria abundance was significantly increased over three years in the Gurbantunggut desert as a response to nitrogen fertilization application. This is consistent with the result of the positive correlation between the relative abundance of soil actinobacteria and PGPR applied in our research. The concentrate of high-temperature pyrolysis biochar has been demonstrated as an extremely easy decomposed carbon source for soil *Actinomyces* [45], which could explain the increase in the relative abundance of soil Actinobacteria in our study. The abundance of Gemmatimonadetes was mainly correlated to soil types and environmental factors, as in most studies [46,47]. Gemmatimonadetes was positively related to soil moisture content, which is consistent with the relationship between SWC significantly affected by biochar and relative abundance of Gemmatimonadetes in our study. Most Cyanobacteria has the potential to biologically fix N with its nifH gene [48]; thus, positive correlation between soil Cyanobacteria abundance and soil N content has been reported in previous studies [49,50], which is in line with the correlation between soil TN significantly influenced by PGPR and relative abundance of Cyanobacteria.

Chloroflexi belongs to the gram-negative bacteria and could potentially autotrophically metabolize through photosynthesis; thus, the growth and reproduction of Chloroflexi do not rely on the soil nutrition supply in terrestrial environments. Calderón et al. [30] reported that soil Chloroflexi abundance showed a positive correlation with soil pH in a reciprocal transplant design experiment, which is consistent with the results of our study. Khodadad et al. [47] reported that applying 20-60 t hm^-2^ biochar may decrease soil Chloroflexi abundance through regulating soil available N, available P, and available K. The decrease in soil Chloroflexi relative abundance after biochar and biochar+PGPR may be a consequence of increased inorganic N content that resulted from biochar application, if sufficient available nitrogen was supplied for plant growth; it thus potentially inhibited the reproduction of Chloroflexi. Soil Firmicutes was reported to increase following biochar amendment [47], which is in line with the increase in the relative abundance of soil Firmicutes after the application of biochar in our study. Koch et al. [51] reported that *Nitrosomonas*, the dominant genus of Nitrospirae, can hydrolyze urease into NH_4_^+^ and CO_2_ in soils under a low concentration of ammonium nitrogen and further increase soil inorganic N. This is consistent with our finding that soil inorganic N was positively correlated with the relative abundance of soil Nitrospirae. Verrucomicrobia is one of the bacteria that takes part in carbon (C) cycling and fixation in acid soil, and in some previous research [52,53,54], Verrucomicrobia were classified as methane fixation bacteria, which potentially transferred methane into CO_2_ or biomass through utilizing the NH_4_^+^-N in soil. These findings are similar to our result that negative correlations were observed between soil NH_4_^+^-N and the relative abundance of Verrucomicrobia following PGPR amendments (both separate application of PGPR and co-application of PGPR and biochar).

### 4.4. Soil Bacterial Community Composition (Genus Level)

Soil pH influences bacterial distribution in terrestrial environments through regulating the microbial habitat environment. Feng et al. [55] reported that soil pH is a key predictor of the structure of soil bacterial communities, which is in line with the positive correlation between the relative abundance of soil Micrococcaceae and soil pH. Nocardioides potentially biologically degrade polycyclic aromatic hydrocarbons (PAHs) and are widely distributed in plant rhizosphere soil [56]. The increase of the relative abundance of Nocardioides in the biochar treatment was mainly because PAHs easily accumulated on the surface of biochar and may stimulate the reproduction of Nocardioides. Anaerolineaceae is the representative bacteria family of Chloroflexi and mainly takes part in the digestion and degradation of organic matter [57], the response trends of Anaerolineaceae were more similar among treatments than Chloroflexi. Sphingomonas belongs to gram-negative bacteria and potentially decomposes organic compounds (especially poly-chlorophenol) in soil, which is similar to the function of Nocardioides in soil ecological environment maintenance. The increases of the relative abundance of *Roseiflexus* in biochar amendments were mainly because *Roseiflexus* is one of the genera of aerobic and thermophilic gram-negative bacteria [58], and biochar applied to soil contributed to the absorption of soil heat and thus promoted the fast growth and reproduction of *Roseiflexus*. Deslippe et al. [59] reported that the abundance of Gemmatimonadaceae was statistically related to soil temperature, which is in line with the significant increases of the relative abundance of Gemmatimonadaceae following biochar amendments in our research. Carotene (i.e., chlorophyll-a and chlorophyll-b) is widely distributed in Rhodospirillaceae cells, which potentially makes Rhodospirillaceae photosynthesis without oxygen-releasing. Lehmann et al. [60] reported that 20–60 t hm^−2^ biochar applied could significantly increase the abundance of soil Rhodospirillaceae, which is consistent with the result that co-application or separate application of biochar increased the relative abundance of Rhodospirillaceae in our study. The relative abundance of Acidimicrobiales was significantly increased by all PGPR treatments (PGPR and biochar+PGPR), the main reason being that the lactic acid produced from the activity of probiotics could be used as the carbon resource of Acidimicrobiales. This is also consistent with the finding of the increased diversity of rhizosphere microbial in soil upon the PGPR amendment [56]. Short-term separate application of biochar and PGPR potentially increased the relative abundance of *Bacillus* in our research, whereas co-application of biochar and PGPR had an inverse impact on them. The probable reason that short-term separate application of biochar and PGPR increased the relative abundance of *Bacillus* and co-application of biochar and PGPR had an inverse impact on it may be that *Bacillus* could uptake and utilize the nutrition from the surface of the biochar and PGPR applied in our research and has been certified to be *Bacillus megaterium*.

### 4.5. Suggestion for Using Biochar and PGPR to Improve Soil Properties and Bacterial Diversity

The significantly positive responses of soil NO_3_^−^-N, inorganic N, EC, and SWC to biochar and biochar+PGPR applications, and soil TN concentration to PGPR and biochar applications, and soil TK concentration to PGPR and biochar+PGPR demonstrated that co-application or separate application of biochar and PGPR is beneficial to specific soil nutrients in the short term, although decrease in soil TP and NH_4_^+^-N in PGPR treatment was also observed. Further, the effects on soil physicochemical properties were significantly influenced by the manner of application (co-application or separate application) of the biochar and PGPR used in our study. Furthermore, the significantly positive responses of the relative abundance of soil Actinobacteria, Gemmatimonadetes, and Cyanobacteria to all of the treatments reflected that co-application or separate application of biochar and PGPR was conducive to a specific soil community, based on the phylum level, although decreases in Proteobacteria, Acidobacteria, and Bacteroidetes were also observed. Acidobacteria has been reported to be a predictor of soil health status and generally negatively correlated to soil quality, especially in relatively barren soil [61]. The significant decrease in Acidobacteria relative abundance in our study indicates that soil quality was improved by the applications of biochar and PGPR. The cluster analysis result showed that the bacterial community in PGPR and biochar+PGPR treatments were apparently different from those of the biochar and control in relation to the genus level, indicating that the co-application of biochar and PGPR changed the soil bacterial community relative to the control and separate application of biochar. The RDA results showed that soil physicochemical properties had no significant impact on the phylum level of soil bacterial composition, whereas soil NH_4_^+^-N significantly influenced the genus level of soil bacterial composition. One limitation of our research was that our study focused only on the relationship between soil bacterial community and soil nutrient contents, but soil nutrient status may also depend on the interaction between plant and soil through nutrient transformations.

## 5. Conclusions

Our study indicates that biochar and PGPR amendments modify soil physicochemical properties in soils in *Eucalyptus* plantations. The co-application of biochar and PGPR significantly increases NO_3_-N, inorganic N, total K, and soil water content, contributing to plant and microbial nitrogen and potassium supply and the improvement of moisture conditions. 

This study also revealed that the manner of application (co- or separate) of biochar and PGPR significantly influences the bacterial community composition by increasing bacterial OTU richness and diversity and increasing the relative abundance of Actinobacteria, Gemmatimonadetes, and Cyanobacteria. 

Results also indicate that soil NH_4_^+^-N might serve as a sensitive indicator of soil bacterial community composition at the genus level, providing useful information on soil microbial activity and ecological stability. 

We encourage the co-application of biochar and PGPR as bio-fertilizer, as it has the potential to reduce the heavy demand for artificial N fertilizer in *Eucalyptus* plantations and enhances soil bacterial diversity.

## Figures and Tables

**Table 1:** Basic properties of biochar in our research. (Fixed C: fixed carbon, Av. P: Olsen available phosphorus; Av. K: available potassium; Bulk: bulk density; SA: surface area; EC: electrical conductivity CEC: cation exchange capacity).

Fixed C (g kg^−1^)	Av.P (g kg^−1^)	Av. K (g kg^−1^)	Bulk (g cm^−3^)	SA (m^2^ g^−1^)	Porosity (%)	pH	EC (mS cm^−1^)	CEC (cmol kg^−1^)
650	10.20	55.65	0.19	9	67.03	10.24	4.68	60.80

**Table 2:** PCR primer of bacteria.

Aimed Object	Primer	Sequence (5’–3’)
Bacterial16S rRNA gene	338F	ACTCCTACGGGAGGCAGCAG
	806R	GGACTACHVGGGTWTCTAAT

**Table 3:** PCR reaction system and amplification program of bacteria.

PCR Reaction System (20 μL)	Addition (μL)	Amplification System
5×FastPfu Buffer	4	Denaturation at 95 ℃ for 3 min
2.5mM dNTPs	2	Degeneration at 95 ℃ for 30 s
Forward Primer (5 µM)	0.8	Annealing at 55 ℃ for 30 s
Reverse Primer (5 µM)	0.8	Extension at 72 ℃ for 45 s
FastPfu Polymerase	0.4	25 recycling
BSA	0.2	Extension at 72 ℃ for 10 min
Template DNA	10 ng	
Add ddH_2_O to	20	Stored at 10 ℃

**Table 4:** Means and standard errors of soil nutrient contents amended with plant growth-promoting rhizobacteria (PGPR) and biochar. Different letters indicate significant differences at *p* < 0.05 among treatments and the control. NO_3_^−^-N: nitrate nitrogen; NH_4_^+^-N: ammonium nitrogen; IN: inorganic nitrogen; TN: total nitrogen; TP: total phosphorus; TK: total potassium; EC: electrical conductivity; SWC: soil water content.

Treatment	NO_3_^−^-N (mg g^−1^)	NH_4_^+^-N (mg g^−1^)	IN (mg g^−1^)	TN (mg g^−1^)	TP (mg g^−1^)	TK(mg g^−1^)	pH	EC (dS m^−1^)	SWC (%)
Control	0.038 ± 0.002 ^c^	0.028 ± 0.001 ^a^	0.066 ± 0.003 ^c^	1.03 ± 0.05 ^c^	2.31 ± 0.12 ^a^	1.45 ± 0.07 ^c^	8.33 ± 0.42 ^ab^	105 ± 5 ^b^	13 ± 1 ^c^
Biochar	0.050 ± 0.003 ^b^	0.025 ± 0.001 ^b^	0.075 ± 0.004 ^b^	1.28 ± 0.06 ^b^	1.99 ± 0.10 ^b^	1.58 ± 0.08 ^c^	7.84 ± 0.39 ^b^	128 ± 6 ^a^	14 ± 1 ^b^
PGPR	0.024 ± 0.001 ^d^	0.025 ± 0.001 ^b^	0.049 ± 0.003 ^d^	2.06 ± 0.10 ^a^	1.86 ± 0.09 ^b^	1.77 ± 0.09 ^b^	8.78 ± 0.44 ^a^	88 ± 4 ^c^	13 ± 1 ^bc^
Biochar+PGPR	0.064 ± 0.003 ^a^	0.025 ± 0.001 ^ab^	0.089 ± 0.005 ^a^	1.06 ± 0.05 ^c^	1.41 ± 0.07 ^c^	2.25 ± 0.11 ^a^	7.89 ± 0.39 ^b^	135 ± 7 ^a^	17 ± 1 ^a^

**Table 5:** Means and standard errors of the number of observed operational taxonomic units (OTUs) (at 97% similarity), richness, diversity, and coverage of soil bacteria. Different lowercase letters showed significant difference at *p* < 0.05 among treatments and the control.

Treatments	Reads	OTUs	Coverage	Richness and Diversity Indices		
				Simpson	ACE	Chao
Control	26784 ± 1339 ^c^	1864 ± 58 ^c^	0.98 ± 0.00 ^b^	0.0032 ± 0.0005 ^d^	2176 ± 26 ^b^	2188 ± 16 ^c^
Biochar	35153 ± 1758 ^b^	2185 ± 71 ^b^	0.99 ± 0.00 ^a^	0.0190 ± 0.0047 ^a^	2615 ± 29 ^a^	2631 ± 1 ^b^
PGPR	41703 ± 2085 ^a^	2408 ± 205 ^a^	0.99 ± 0.00 ^a^	0.0066 ± 0.0005 ^c^	2714 ± 211 ^a^	2696 ± 277 ^ab^
Biochar+PGPR	35036 ± 1752 ^b^	2324 ± 1 ^a^	0.99 ± 0.01 ^ab^	0.0107 ± 0.0008 ^b^	2721 ± 110 ^a^	2767 ± 135 ^a^

## Supplementary Materials

The following are available online at https://www.mdpi.com/2076-2607/8/4/502/s1: Equation S1: algorithm used for the calculation of Chao1. Equations S2, S3, S4 and S5: algorithms used for the calculation of ACE. Equation S6: algorithm used for the calculation of Simpson index.

## Abbreviations

TK: total potassium; TN: total nitrogen; TP: total phosphorus; NH_4_^+^-N: ammonium nitrogen; NO_3_^-^-N: nitrate nitrogen; IN: inorganic nitrogen; SWC: soil water content; EC: electrical conductivity; PGPR: plant growth-promoting rhizobacteria; NCBI: National Center for Biotechnology Information; OTU: operational taxonomic units; PCR: polymerase chain reaction; BSA: Albumin from bovine serum.
