# Supplementary material for: Biochar and Rhizobacteria Amendments Improve Several Soil Properties and Bacterial Diversity
Source: Microorganisms. 2020 Apr 1;8(4):502. doi: 10.3390/microorganisms8040502 (PMC7232174; doi:10.3390/microorganisms8040502)
Supplement: Supplementary file 1 [file microorganisms-08-00502-s001.pdf]

Equation S1:

$$\mathrm{Schao1}=S_{obs}+\frac{n_1(n_1-1)}{2(n_2+1)}$$

Where S_{chao1} is the estimated number of OTU, S_{obs} is the number of OTU from actual observation, n_1 is the number of OTU with only one sequence (i.e. singletons), n_2 is the number of OTU with two sequences (i.e. doubletons).

Equations S2,S3,S4,S5:

$$S_{ACE}=\begin{cases} S_{abund}+\frac{S_{rare}}{C_{ACE}}+\frac{n_1}{C_{ACE}}\hat{\gamma}^2_{ACE}, \text{ for } \&\hat{\gamma}_{ACE}<0.80 \\ S_{abund}+\frac{S_{rare}}{C_{ACE}}+\frac{n_1}{C_{ACE}}\tilde{\gamma}^2_{ACE}, \text{ for } \&\hat{\gamma}_{ACE}\geq 0.80 \end{cases}$$

$$N_{rare}=\sum_{i-1}^{abund} in_i, \quad C_{ACE}=1-\frac{n_1}{N_{rare}}$$

$$\hat{\gamma}^2_{ACE}=\max\left[\frac{S_{rare}}{C_{ACE}}\frac{\sum_{i-1}^{abund} i(i-1)n_i}{N_{rare}（N_{rare}-1）}-1,0\right]$$

$$\tilde{\gamma}^2_{ACE}=\max\left[\hat{\gamma}^2_{ACE}\left\{1+\frac{N_{rare}(1-C_{ACE})\sum_{i-1}^{abund} i(i-1)n_i}{N_{rare}（N_{rare}-C_{ACE}）}\right\},0\right]$$

Where n_i is the number of OTU with i sequences, S_{rare} is the number of OTU with or less that "abund" sequences, S_{abund} is the number of OTU more than "abund" sequences, abund is the threshold of dominate OTU and default to be 10.

Equation S6:

$$D_{simpson}=\frac{\sum_{i=1}^{S_{obs}} n_i(n_i-1)}{N(N-1)}$$

Where S_{obs} is the number of OTU from actual observation, n_i is the number of sequences of i- th OTU, N is the number of all sequences.
